# Transient Global Amnesia After Screening Esophagogastroduodenoscopy: Incidence and Risk Factors in a 20‐Year Single‐Center Cohort

**DOI:** 10.1002/deo2.70298

**Published:** 2026-02-10

**Authors:** Miyuki Kobayashi, Ichiro Takayama, Masahiko Ohtaka, Yasuaki Ishida, Yu Hihara, Mika Miura, Kenji Hosoda, Yuichi Hirose, Kazuhiko Takaso, Yoshioki Yoda, Fumikazu Kobayashi, Hiroshi Yokomichi, Nobuyuki Enomoto

**Affiliations:** ^1^ Yamanashi Koseiren Health Care Center Yamanashi Japan; ^2^ Health Care Center University of Yamanashi Yamanashi Japan; ^3^ Department of Neurology Koshu Rehabilitation Hospital Yamanashi Japan; ^4^ Department of Epidemiology and Environmental Medicine University of Yamanashi Yamanashi Japan; ^5^ Department of Gastroenterology and Hepatology Faculty of Medicine University of Yamanashi Yamanashi Japan

**Keywords:** esophagogastroduodenoscopy, risk factors, screening endoscopy, transient global amnesia, unsedated endoscopy

## Abstract

**Background and Aim:**

Transient global amnesia (TGA) after esophagogastroduodenoscopy (EGD) has been described only in case reports. Because sudden‐onset amnesia can mimic stroke and other acute central nervous system disorders, TGA requires careful differentiation in the acute setting, and clinicians need to be familiar with this condition. We estimated its incidence after screening EGD and identified associated risk factors in a large single‐center cohort.

**Methods:**

We retrospectively reviewed 420,979 screening EGDs at a single health checkup center (April 2005–March 2025). All EGDs were performed without sedation, using topical pharyngeal anesthesia only. TGA cases were ascertained from the endoscopy adverse‐event registry and confirmed. For analyses, three non‐TGA controls per TGA case (1:3 ratio) were randomly selected from examinees undergoing EGD on the same calendar date, using same‐day matching. Odds ratios (ORs) were estimated using conditional logistic regression.

**Results:**

TGA occurred in 64 episodes in 63 individuals of 420,979 examinations (0.0152%). In route‐specific analyses from April 2008, the transoral route carried a higher risk than the transnasal route (Fisher's exact test *p* = 0.013). In multivariable matched models, older age (odds ratio [OR] 2.32 per 10‐year increase; 95% confidence interval [CI], 1.38–3.92), female sex (14.6; 4.82–43.9), transoral insertion (10.2; 1.88–55.4), and treated dyslipidemia (2.84; 1.20–6.73) were statistically associated. EGD time and endoscopist experience were not associated.

**Conclusions:**

The incidence of post‐EGD TGA was very low at 15.2 per 100,000 examinations (0.0152%). Older age, female sex, transoral insertion, and treated dyslipidemia were independently associated with post‐EGD TGA. A transnasal approach might be a potential risk‐reduction strategy.

## Graphical abstract

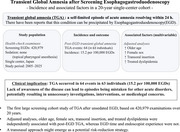

## 1 Introduction

Transient global amnesia (TGA) is a syndrome defined by temporary memory loss without disturbance of consciousness or focal neurological signs [1, 2]. The principal symptom is the sudden onset of impaired memory formation, in which patients are unable to retain new information and repeatedly forget events that occurred only minutes earlier (anterograde amnesia) [1, 2]. During the episode, the retention span for new information is reduced to 30–180 s [2]. Although memory for events occurring during the episode is impaired, symptoms typically resolve within 24 h [1, 2]. In addition, patients often exhibit retrograde amnesia, with memory loss extending back to events that occurred prior to the onset [1, 2]. TGA is a disorder that occurs predominantly between 50 and 70 years of age [2]. Various physical and psychological stressors and Valsalva‐like maneuvers have been reported as triggers, but the cause of TGA remains unknown [2]. Esophagogastroduodenoscopy (EGD) has also been reported as a potential trigger. However, the published evidence is limited to case reports [3, 4, 5, 6, 7, 8, 9] despite its clinical importance. Because the sudden‐onset amnesia of TGA can mimic stroke (including transient ischemic attack) and other acute central nervous system disorders, careful differentiation is required to avoid severe anxiety in patients and families and potentially unnecessary interventions or medicolegal concerns. In clinical practice, post‐EGD TGA could be alarming: patients might repeatedly ask the same questions and appear briefly panicked or confused. In the screening of healthy individuals, this sudden change could also provoke marked anxiety or panic in accompanying family members. In a 20‐year single‐center cohort of unsedated screening EGDs, we therefore estimated the incidence of post‐EGD TGA and identified associated risk factors.

## 2 Methods

### 2.1 Study Design and Setting

This was a retrospective cohort study conducted at the Yamanashi Koseiren Health Care Center. We reviewed all screening EGDs performed between April 2005 and March 2025. All EGDs were performed without sedation, using topical pharyngeal anesthesia only.

### 2.2 Participants

The study population comprised 420,979 screening EGD examinations performed during the study period. The annual mean age of examinees gradually increased from 53.3 years in 2005 to 60.2 years in 2024, corresponding to an average increase of approximately 0.35 years per year on linear regression. Route‐specific incidence was evaluated in the subset of examinations performed between April 2008 and March 2025, when both transoral and transnasal routes were concurrently available.

### 2.3 Identification of TGA Cases

TGA cases were identified from our endoscopy adverse‐event registry and verified. TGA was suspected when patients developed acute memory loss after EGD, often recognized by staff when the patient was seen wandering around the unit and repeatedly asking the same question [9]. Initial assessment was performed by an in‐house physician, followed by a brain magnetic resonance imaging (MRI) to exclude acute cerebrovascular disease. In all cases in which MRI was performed at our center, no acute intracranial abnormalities were identified. Patients were then referred to neurology or neurosurgery specialists for confirmation. All cases fulfilled the Hodges‐Warlow diagnostic criteria [2]. (i) Acute onset and pronounced new memory impairment. (ii) Duration of at least 1 h, regression within 24 h. (iii) No focal neurological symptoms/deficits and no additional cognitive deficits. (iv) Absence of impaired consciousness or disorientation in the person. (v) No previous trauma or epilepsy [2].

### 2.4 Control Selection

For each post‐EGD TGA case, we constructed a same‐day risk set comprising all examinees who underwent screening EGD at our center on the same calendar day and did not meet the TGA case definition. Within each risk set, we randomly selected three non‐TGA controls by assigning a uniform random number to each eligible examinee using Microsoft Excel's random‐number generator (RAND), sorting by the random number, and choosing the first three without replacement. Conditioning on the index calendar date (i.e., same‐day matching) was intended to reduce confounding by temporal factors, including calendar date and day‐of‐week (e.g., variation in staffing patterns and daily case mix). No additional matching beyond same‐day matching was imposed a priori. A 1:3 case–control ratio was chosen to increase statistical precision under fixed resources while maintaining feasibility. We additionally verified that no individual was selected as a control more than once across matched sets.

### 2.5 Variables and Definitions

We evaluated age, sex, insertion route (transoral/ transnasal), EGD time (scope‐in to withdrawal), endoscopist experience (years), and comorbidities (hypertension, diabetes, dyslipidemia—captured in the database as “under treatment”). Age, EGD time, and endoscopist experience were modeled as continuous variables.

### 2.6 Statistical Analysis

Analyses were conducted using EZR (v1.68) [10]. Group differences were assessed using Welch's t‐tests for continuous variables and Fisher's exact test or the chi‐square test for categorical variables, as appropriate. For risk‐factor analyses, we performed a same‐day matched case–control analysis and estimated odds ratios using conditional logistic regression stratified by matched set; age, EGD time, and endoscopist experience were entered as continuous variables. Two‐sided *p*‐values <0.05 were considered statistically significant.

## 3 Results

**FIGURE 1:**
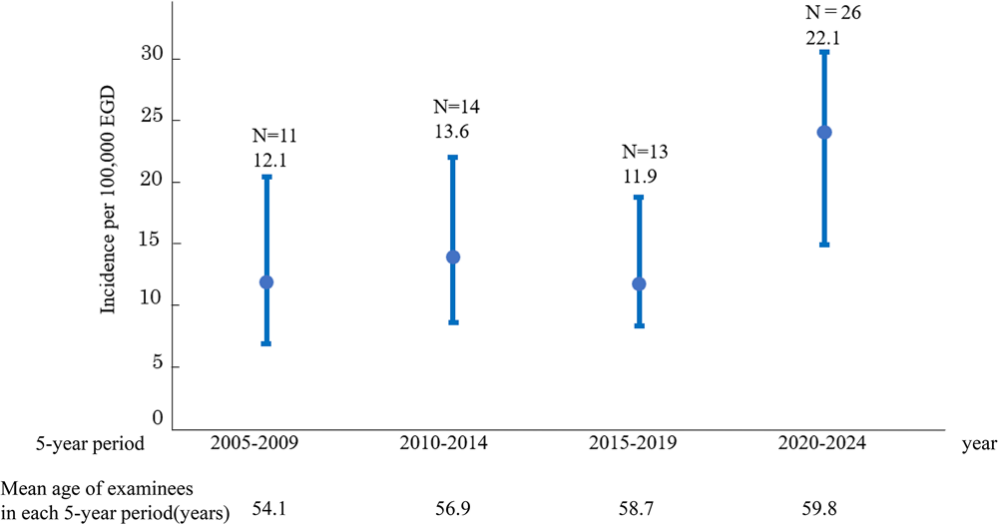
Incidence of post–esophagogastroduodenoscopy (EGD) transient global amnesia (TGA) across 5‐year periods. Bars show the incidence per 100,000 EGDs; whiskers indicate exact Poisson (Garwood) 95% confidence intervals [CIs]. The incidence was 12.1 (95% CI, 6.0–21.6) in 2005–2009, 13.6 (95% CI, 7.4–22.8) in 2010–2014, 11.9 (95% CI, 6.4–20.4) in 2015–2019, and 22.1 (95% CI, 14.4–32.3) in 2020–2024. No statistically significant differences in incidence were observed between any 5‐year periods. The mean age of health‐check examinees undergoing EGD in each 5‐year period is shown on the lower part of the graph (54.1 years in 2005–2009, 56.9 in 2010–2014, 58.7 in 2015–2019, and 59.8 in 2020–2024).

Among 420,979 screening EGDs, 64 TGA events in 63 individuals were identified (15.2 per 100,000 EGDs; equivalent to 0.0152%). When stratified by 5‐year period, incidence was 12.1 per 100,000 EGDs (11/91,185; 2005–2009), 13.6 per 100,000 EGDs (14/103,007; 2010–2014), 11.9 per 100,000 EGDs (13/108,963; 2015–2019), and 22.1 per 100,000 EGDs (26/117,824; 2020–2024). No statistically significant differences in incidence were observed between any 5‐year periods (Figure 1).

**FIGURE 2:**
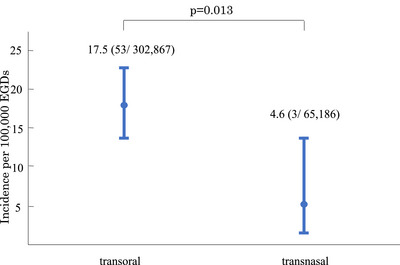
Incidence of transient global amnesia (TGA) after esophagogastroduodenoscopy (EGD) per 100,000 EGDs by insertion route (April 2008–March 2025). Bars show route‐specific incidence; whiskers indicate exact Poisson (Garwood) 95% confidence intervals [CIs]: transoral 17.5 (95% CI, 13.1–22.9) and transnasal 4.6 (95% CI, 0.9–13.5). Numbers above bars indicate the incidence and counts (events/total). The proportion difference was significant by two‐sided Fisher's exact test (p = 0.013).

Among the 64 post‐EGD TGA events (63 individuals), transoral insertion was used in 61 and transnasal in three. Because the transnasal route was introduced in 2008, route‐specific incidence was calculated for April 2008–March 2025: 53 TGA events occurred after 302,867 transoral EGDs (17.5 per 100,000; 95% confidence interval [CI], 13.1–22.9) versus three after 65,186 transnasal EGDs (4.6 per 100,000; 95% CI, 0.9–13.5), (*p* = 0.013, Figure 2).

We identified 64 post‐EGD TGA episodes in 63 individuals, including one recurrent post‐EGD episode. The individuals had a mean age of 65.1 ± 6.5 years, and 90.5% (57/63) were female. Mean EGD time was 322.7 ± 176.0 s and mean endoscopist experience was 20.4 ± 12.9 years. Treated dyslipidemia was present in 24 patients (38.1%). TGA recurred in three individuals (3/63, 4.8%). One recurrence occurred after a subsequent EGD at our center, whereas the other two occurred in non‐endoscopic settings. Although a persistent memory gap remained for the period of amnesia during the TGA episode, no other neurologic deficits or clinically significant sequelae were observed.

Compared with non‐TGA controls, TGA cases were significantly older and had higher frequencies of female sex, transoral insertion, and treated dyslipidemia (Table 1). Because one examinee experienced two post‐EGD TGA episodes, we included only one index episode per individual (the first episode) in the matched analyses to avoid non‐independence, yielding 63 matched sets and 189 controls (1:3).

**TABLE 1:** Characteristics between cases with and without transient global amnesia (TGA) after esophagogastroduodenoscopy (EGD).

	TGA index cases	Non‐TGA cases	*p*‐value
Cases, *n*	63	189	
Age, years (range)	65.1 ± 6.5(50–79)	57.9 ± 12.2(28–88)	**<0.001**
Female:Male, *n*	57:6	93:96	**<0.001**
Transoral:Transnasal, *n*	60:3	154:35	**<0.001**
EGD time, seconds	322.7 ± 176.0	284.4 ± 126.5	0.114
Endoscopist experience, years	20.4 ± 12.9	20.3 ± 12.0	0.957
Dyslipidemia, *n* (%)	24 (38.1)	34 (18.0)	**<0.001**
Hypertension, *n* (%)	16 (25.4)	61 (32.3)	0.346
Diabetes, *n* (%)	5 (7.9)	11 (5.8)	0.556

Values are *n* **(%)** or mean ± SD (range); SD, standard deviation. Continuous variables were compared using Welch's t‐test; categorical variables were compared using Fisher's exact test.

In multivariable matched conditional logistic regression models, older age (odds ratio [OR] 2.32 per 10‐year increase, 95% CI 1.38–3.92; *p* = 0.002), female sex (OR 14.6, 95% CI 4.82–43.9; *p* < 0.001), transoral insertion (OR 10.2, 95% CI 1.88–55.4; *p* = 0.007), and treated dyslipidemia (OR 2.84, 95% CI 1.20–6.73; *p* = 0.017) remained independently associated with post‐EGD TGA. Endoscopist experience (OR 1.00 per 1‐year increase, 95% CI 0.97–1.03; *p* = 0.949) and EGD time (OR 1.13 per 1‐minute increase, 95% CI 1.00‐1.29; *p* = 0.055) were not significantly associated (Table 2).

**TABLE 2:** Risk factors for transient global amnesia (TGA) after esophagogastroduodenoscopy (EGD).

	TGA (*n* = 63)	Non‐TGA (*n* = 189)	Univariate analysis			Multivariable analysis		
			OR	95% CI	*p*‐value	OR	95% CI	*p*‐value
Age, years	65.1 ± 6.5	57.9 ± 12.2	2.21	1.51–3.20	**<0.001**	2.32	1.38–3.92	**0.002**
Female sex, *n*	58	93	8.77	3.66–21.0	**<0.001**	14.60	4.82–43.9	**<0.001**
Transoral insertion, *n*	60	154	4.88	1.42–16.7	**0.012**	10.20	1.88–55.4	**0.007**
EGD time, seconds	322.7 ± 176.0	284.4 ± 126.5	1.13	1.00–1.29	0.055	—	—	—
Endoscopist experience, years	20.4 ± 12.9	20.3 ± 12.0	1.00	0.97–1.03	0.949	—	—	—
Dyslipidemia, *n*	24	34	3.15	1.58–6.29	**0.001**	2.84	1.20–6.73	**0.017**
Hypertension, *n*	16	61	0.68	0.34–1.35	0.272	—	—	—
Diabetes, *n*	5	11	1.41	0.46–4.29	0.548	—	—	—

Age: per 10‐year increase.

EGD time: per 1‐min increase.

Endoscopist experience: per 1‐year increase.

Univariable and multivariable ORs were estimated using matched conditional logistic regression.

## 4 Discussion

We observed 64 post‐EGD TGA episodes in 63 individuals in a large screening EGD program. We begin by summarizing the typical clinical presentation and time course of post‐EGD TGA as encountered in our practice [9]. In our clinical practice, TGA was suspected when examinees developed sudden‐onset amnesia after EGD. The affected examinees proceeded with and completed subsequent same‐day examinations after EGD (e.g., vision testing, fundus photography, and hearing tests) without apparent difficulty. Consequently, episodes were often first recognized outside the endoscopy unit: approximately 1–2 h after EGD, non‐endoscopy staff noticed atypical behavior such as pacing or wandering and repetitive questioning (e.g., “Why am I here?”). Although patients appeared to understand an explanation briefly, they quickly repeated the same questions, a pattern strongly suggestive of acute anterograde amnesia. The clinical course was characterized by anterograde amnesia lasting a mean of 2.8 h (range, 1–6), accompanied by retrograde amnesia affecting a mean of 2 h (range, 1–4), durations that were somewhat shorter than those generally reported for TGA [9]. After the anterograde amnesia resolved (with cessation of repetitive questioning), patients had a persistent memory gap for the episode, with no recovery of memories formed during the amnestic period, although retrograde amnesia sometimes partially improved. No other neurologic deficits or clinically meaningful sequelae were observed. At our center, TGA was suspected clinically, and affected examinees were referred to a neurosurgical hospital for specialist evaluation and confirmation of the final diagnosis; in all cases, the final diagnosis remained TGA, and no alternative diagnosis was established [9].

In our health checkup program, in addition to EGD, spirometry and abdominal ultrasonography are routinely performed. Chest computed tomography and brain MRI can also be conducted on the same day upon request. Among these examinations, only spirometry has been reported once as a potential trigger of TGA [11], apart from EGD. However, even during the COVID‐19 pandemic (2020–2022), when spirometry was suspended, 11 post‐EGD TGA episodes occurred. The incidence of TGA per 100,000 EGDs during these three years did not differ from that in the remaining 17 years (11/69,767 vs. 53/351,212 examinations; *p* = 0.89). Moreover, no clinically confirmed TGA cases were identified among health‐check examinees who did not undergo EGD. Taken together, although causality cannot be definitively established in this observational study, the consistent temporal proximity to EGD, the fact that EGD was the only exposure shared by all cases, and the absence of other plausible triggers suggest that screening for EGD might have acted as the precipitating factor for TGA in this cohort.

During the study period, 420,979 unsedated screening EGDs were performed, and TGA occurred at a rate of 15.2 per 100,000 examinations (0.0152%). Although the incidence varied numerically across 5‐year periods, these differences were not statistically significant and should be interpreted cautiously. Such fluctuations might reflect random variation given the small number of events and/or changes over time in case ascertainment (staff awareness), the age distribution of examinees, and endoscopic practice (including examination time).

In adjusted analyses, older age, female sex, transoral insertion, and treated dyslipidemia were independently associated with post‐EGD TGA, whereas EGD time and endoscopist experience were not. In previously published case reports [3, 4, 5, 6, 7, 8, 9], post‐EGD TGA mainly affected middle‐aged or older women. In contrast, current guidelines state that general (non‐EGD) TGA occurs with approximately equal frequency in men and women [2]. In women with general TGA, episodes have been reported to be frequently associated with emotional precipitating events, a history of anxiety, and certain personality traits [2, 12]. These observations raise the possibility that heightened anxiety or fear toward endoscopy might contribute to the female predominance observed in post‐EGD TGA [9]. In this report, the incidence was lower with transnasal EGD when both routes were available. Given the few transnasal events and potential residual confounding, these findings should be interpreted cautiously. A transnasal option might be considered for examinees who prefer it (e.g., those with heightened endoscopy‐related anxiety or fear, including some middle‐aged or older women), but whether it reduces post‐EGD TGA requires confirmation in larger studies.

Treated dyslipidemia was associated with post‐EGD TGA in our cohort. One case–control study reported higher rates of hyperlipidemia and ischemic heart disease in patients with TGA, suggesting a possible vascular contribution [13]. However, other studies have not shown consistent associations with cardiovascular risk factors or clustering of cerebral infarcts, and current guidelines consider cerebral ischemia an unlikely cause of TGA [2]. Furthermore, the guidelines state that TGA has not been convincingly demonstrated to result from arterio‐arterial or cardiac embolism, and that patients do not have an increased risk of subsequent stroke [2]. A recent study also linked atorvastatin to memory loss, including TGA [14]. Thus, both dyslipidemia itself and its treatment might be relevant to TGA risk. However, because detailed information on lipid‐lowering therapy (drug class, dose, and treatment duration) was unavailable, these mechanisms could not be examined in our cohort, and causality cannot be inferred.

Regarding recurrence, one of the 63 patients experienced TGA twice after EGD at our center (1.5%). When medical history was obtained by interview, two additional patients reported a previous TGA episode that was triggered outside of endoscopy. In total, three patients (4.8%) had recurrent TGA. By contrast, guidelines for general (non‐EGD) TGA report a recurrence risk of 12–27% over the disease course [2]. The lower recurrence rate in this report might reflect the lesser influence of the endoscopy procedure itself as a precipitating factor for TGA.

To our knowledge, this is the largest and first cohort study focusing on TGA after unsedated screening EGD. Although rare, post‐EGD TGA is a phenomenon that may be encountered in large screening programs. When TGA actually occurs, lack of awareness of the condition can lead to episodes being mistaken for other acute disorders, potentially resulting in unnecessary investigations, interventions, or medicolegal concerns. For endoscopists, recognizing this characteristic presentation is essential. Such awareness can help reduce undue concern for both clinicians and patients and facilitate appropriate patient counseling.

Limitations include the single‐center, retrospective design and the 20‐year study span, during which techniques evolved, and some procedures may not reflect current standards. External validity is limited to unsedated screening EGD, limiting generalizability to symptomatic populations, hospital settings, sedated EGD, and populations outside Japan. Residual confounding cannot be excluded. Lipid‐lowering therapy was recorded only as “treated dyslipidemia,” without information on drug class, dose, or duration.

## 5 Conclusions

We conducted the first large screening cohort study to investigate TGA following unsedated EGD. The incidence was very low at 15.2 per 100,000 examinations (0.0152%). Older age, female sex, transoral insertion, and treated dyslipidemia were independently associated with post‐EGD TGA. By contrast, EGD time and endoscopist experience were not significantly associated. A transnasal approach might emerge as a potential risk‐reduction strategy.

## Author Contributions

**Miyuki Kobayashi**: conceptualization, study design, data acquisition, formal analysis, visualization, and writing – original draft. **Ichiro Takayama**: conceptualization, study design, and supervision. **Masahiko Ohtaka**: resources and supervision, conceptualization, project administration, and writing – review & editing. **Yasuaki Ishida**, **Yu Hihara**, **Mika Miura**, **Kenji Hosoda**, **Yuichi Hirose**, and **Kazuhiko Takaso**: investigation and data curation. **Fumikazu Kobayashi**: validation. **Hiroshi Yokomichi**: methodology (statistics), formal analysis, and validation. **Nobuyuki Enomoto**: supervision.

## Funding

No specific funding was received.

## Ethics Statement

**Approval of the research protocol**: The study protocol was approved by the Institutional Review Board of Yamanashi Koseiren Health Care Center (Approval No. 6‐2). The study was conducted in accordance with the Declaration of Helsinki.

## Consent

Informed consent was obtained using an opt‐out approach via information disclosed on our institutional website; the requirement for written consent was waived by the Board.

## Conflicts of Interest

The authors declare no conflicts of interest.

## Clinical Trial Registration

N/A.

## Data Availability

Deidentified data and analysis code are available from the corresponding author upon reasonable request and IRB approval; individual‐level data cannot be shared publicly.

## References

[1] J. R. Hodges and C. P. Warlow , “Syndromes of Transient Amnesia: Towards a Classification,” Journal of Neurology, Neurosurgery, and Psychiatry 53, no. 10 (1990): 834–843, 10.1136/jnnp.53.10.834. 2266362 PMC488242

[2] D. Sander , T. Bartsch , F. Connolly , et al., “Guideline “Transient Global Amnesia (TGA)” of the German Society of Neurology (Deutsche Gesellschaft für Neurologie): S1‐Guideline,” Neurological Research and Practice 5 (2023): 15, 10.1186/s42466-023-00240-0. 37076927 PMC10116751

[3] H. Joshita , M. Enomoto , T. Kihira , et al., “Transient Global Amnesia After Upper Gastrointestinal Endoscopy: A Case Report,” Gastroenterological Endoscopy 32, no. 3 (1990): 589–592, 10.11280/gee1973b.32.589.

[4] K. Tsukada , C. Miyabayashi , K. Furukawa , et al., “Three Cases of Transient Global Amnesia Following Upper Gastrointestinal Endoscopy,” Gastroenterological Endoscopy 48, no. 6 (2006): 1215–1220, 10.11280/gee1973b.48.1215.

[5] H. Naka , “Transient Global Amnesia After Upper Gastrointestinal Endoscopy: A Case Report,” Medical Journal of Hokkaido Association of Medical Service for Workers 24 (1997): 29–31.

[6] Y. Sawada , M. Kamihira , R. Hirakawa , Y. Yoshida , and M. Imawari , “Transient Global Amnesia Presumably Induced by Upper Gastrointestinal Endoscopy: A Case Report,” Progress of Digestive Endoscopy 60, no. 2 (2002): 44–46, 10.11641/pde.60.2_44.

[7] A. Hiraga and T. Matsunaga , “Transient Global Amnesia After Gastroscopy,” Journal of Neurology, Neurosurgery, and Psychiatry 77, no. 8 (2006): 995–996, 10.1136/jnnp.2006.087775. 16844960 PMC2077641

[8] A. Sayilir , M. Kurt , M. Ibis , M. Kekilli , I. K. Onal , and N. Sasmaz , “Transient Global Amnesia Following Upper Gastrointestinal Endoscopy Without Premedication,” Gastroenterology Nursing 32, no. 5 (2009): 362, 10.1097/SGA.0b013e3181b90111. 19820444

[9] M. Kobayashi , I. Takayama , K. Takaso , et al., “Transient Global Amnesia After Opportunistic Screening Esophagogastroduodenoscopy: A Case‐series Analysis,” J Gastrointestinal Cancer Screen 63, no. 2 (2025): 112–119, 10.11404/jsgcs.24021.

[10] Y. Kanda , “Investigation of the Freely Available Easy‐to‐use Software , “EZR” for Medical Statistics,” Bone Marrow Transplantation 48, no. 3 (2013): 452–458, 10.1038/bmt.2012.244. 23208313 PMC3590441

[11] J. C. Williamson and A. J. Larner , “Confused After Spirometry: A Unifying Diagnosis,” BMJ Case Reports 2016 (2016): bcr2016216645, 10.1136/bcr-2016-216645. PMC5129178 27856531

[12] P. Quinette , B. Guillery‐Girard , J. Dayan , et al., “What Does Transient Global Amnesia Really Mean? Review of the Literature and Thorough Study of 142 Cases,” Brain 129, no. 7 (2006): 1640–1658, 10.1093/brain/awl105. 16670178

[13] J. W. Jang , S. Y. Park , J. H. Hong , Y. H. Park , J. E. Kim , and S. Kim , “Different Risk‐factor Profiles Between Transient Global Amnesia and Transient Ischemic Attack: A Large Case‐control Study,” European Neurology 71, no. 1‐2 (2014): 19–24, 10.1159/000354023. 24281363

[14] K. Chen , Y. Chen , and H. Huang , “Exploring the Relationship Between Atorvastatin and Memory Loss: Real‐world Pharmacovigilance and Mendelian Randomization,” Drugs in R&D 24, no. 2 (2024): 317–329, 10.1007/s40268-024-00474-6. 38963511 PMC11315864

